# Cereal and nonfat milk support muscle recovery following exercise

**DOI:** 10.1186/1550-2783-6-11

**Published:** 2009-05-14

**Authors:** Lynne Kammer, Zhenping Ding, Bei Wang, Daiske Hara, Yi-Hung Liao, John L Ivy

**Affiliations:** 1 Exercise Physiology and Metabolism Laboratory Department of Kinesiology and Health Education The University of Texas at Austin Austin, TX, USA

## Abstract

**Background:**

This study compared the effects of ingesting cereal and nonfat milk (Cereal) and a carbohydrate-electrolyte sports drink (Drink) immediately following endurance exercise on muscle glycogen synthesis and the phosphorylation state of proteins controlling protein synthesis: Akt, mTOR, rpS6 and eIF4E.

**Methods:**

Trained cyclists or triathletes (8 male: 28.0 ± 1.6 yrs, 1.8 ± 0.0 m, 75.4 ± 3.2 kg, 61.0 ± 1.6 ml O_2_•kg^-1^•min^-1^; 4 female: 25.3 ± 1.7 yrs, 1.7 ± 0.0 m, 66.9 ± 4.6 kg, 46.4 ± 1.2 mlO_2_•kg^-1^•min^-1^) completed two randomly-ordered trials serving as their own controls. After 2 hours of cycling at 60–65% VO_2MAX_, a biopsy from the vastus lateralis was obtained (Post0), then subjects consumed either Drink (78.5 g carbohydrate) or Cereal (77 g carbohydrate, 19.5 g protein and 2.7 g fat). Blood was drawn before and at the end of exercise, and at 15, 30 and 60 minutes after treatment. A second biopsy was taken 60 minutes after supplementation (Post60). Differences within and between treatments were tested using repeated measures ANOVA.

**Results:**

At Post60, blood glucose was similar between treatments (Drink 6.1 ± 0.3, Cereal 5.6 ± 0.2 mmol/L, p < .05), but after Cereal, plasma insulin was significantly higher (Drink 123.1 ± 11.8, Cereal 191.0 ± 12.3 pmol/L, p < .05), and plasma lactate significantly lower (Drink 1.4 ± 0.1, Cereal 1.00 ± 0.1 mmol/L, p < .05). Except for higher phosphorylation of mTOR after Cereal, glycogen and muscle proteins were not statistically different between treatments. Significant Post0 to Post60 changes occurred in glycogen (Drink 52.4 ± 7.0 to 58.6 ± 6.9, Cereal 58.7 ± 9.6 to 66.0 ± 10.0 μmol/g, p < .05) and rpS6 (Drink 17.9 ± 2.5 to 35.2 ± 4.9, Cereal 18.6 ± 2.2 to 35.4 ± 4.4 %Std, p < .05) for each treatment, but only Cereal significantly affected glycogen synthase (Drink 66.6 ± 6.9 to 64.9 ± 6.9, Cereal 61.1 ± 8.0 to 54.2 ± 7.2%Std, p < .05), Akt (Drink 57.9 ± 3.2 to 55.7 ± 3.1, Cereal 53.2 ± 4.1 to 60.5 ± 3.7 %Std, p < .05) and mTOR (Drink 28.7 ± 4.4 to 35.4 ± 4.5, Cereal 23.0 ± 3.1 to 42.2 ± 2.5 %Std, p < .05). eIF4E was unchanged after both treatments.

**Conclusion:**

These results suggest that Cereal is as good as a commercially-available sports drink in initiating post-exercise muscle recovery.

## Background

Endurance exercise affects skeletal muscle by reducing energy stores and increasing muscle protein breakdown. Although a small amount of glycogen is stored in the liver, the primary energy source during endurance exercise is glycogen stored in skeletal muscle [1]. Exercise duration and intensity can be limited by glycogen availability, emphasizing the importance of replenishing these energy stores prior to subsequent exercise bouts [2]. Exercise also increases muscle protein degradation. Muscle protein breakdown occurs continually, even at rest, releasing amino acids into the intracellular fluid and bloodstream to be used for protein synthesis or oxidized for energy [3-5]. Protein synthesis is stimulated by exercise, but consumption of food must offset breakdown to create a positive net muscle protein balance [6,7].

Following exercise, acute physiological changes occur in the muscle that promote glucose uptake, glycogen accumulation and protein synthesis [6,8,9], but optimal replenishment of the energy stores and net protein balance are dependent on post exercise nutritional content and timing [10-12]. While glycogen synthesis requires glucose, protein synthesis requires amino acids. Combining carbohydrate with protein increases stimulation of the insulin-signaling and mTOR pathways, increasing both glycogen and protein synthesis [13-15], suggesting that the ideal recovery food must contain both carbohydrate and protein to provide substrate for glycogen synthesis and achieve net protein balance.

In addition to the composition of the post-exercise food, exercise duration, intensity and training status influence glycogen and skeletal muscle protein status [1,16-19]. While many exercise protocols used in research are designed to clearly observe post supplementation glycogen and muscle protein changes, these protocols are not typical training sessions for most individuals. For example, glycogen synthesis rate and amount are maximized when subjects exercise to exhaustion to deplete glycogen stores prior to supplementation [1,18,19]. Similarly, protein breakdown and subsequent synthesis is acutely higher after resistance exercise and supplementation in untrained compared to trained subjects [17]. Protocols including a more realistic training scenario and foods such as cereal and nonfat milk may be equally effective in observing responses to post exercise supplementation as compared to using exhaustive protocols or untrained subjects.

Although muscle response during recovery to a carbohydrate-protein drink may be similar to that seen after whole-grain cereal and nonfat milk, we chose to compare a carbohydrate-only drink. Recreational athletes may be more familiar with carbohydrate drinks due to high product awareness and accessibility, and may not understand the benefit of added protein in post-exercise supplementation. Our goals were to use ordinary foods after moderate exercise to understand relative effects on glycogen repletion, and the phosphorylation state of proteins controlling protein synthesis for the average individual. Cereal and milk were selected since both are readily available, popular foods that are inexpensive and easily digested. Our hypothesis was that cereal and nonfat milk would be more effective than a popular carbohydrate-electrolyte sports drink in increasing muscle glycogen and the signaling activity of proteins controlling protein synthesis after moderate endurance exercise.

## Methods

### Participants

Twelve healthy cyclists or triathletes (8 male, 4 female) (Table 1) from the Austin, TX area were recruited via an email announcement to participate in the study. Each volunteer completed a health questionnaire to exclude participants at risk for or with preexisting cardiovascular disease, diabetes or other high-risk medical conditions. Volunteers could not be taking regular medications except for allergy and/or birth-control medicines. Volunteers then reviewed the study protocol and had an opportunity to ask questions prior to signing an informed consent form. The University of Texas at Austin Institutional Review Board for the Protection of Human Subjects approved the study protocol, informed consent form and health questionnaire.

**Table 1:** Subject characteristics, M ± SEM

	**Male (N = 8)**	**Female (N = 4)**
Training Background	7 Cyclists 1 Triathlete	1 Cyclist 3 Triathletes

Age (yrs)	28.0 ± 1.6	25.3 ± 1.7

Height (m)	1.8 ± 0.0	1.7 ± 0.0

Weight (kg)	75.4 ± 3.2	66.9 ± 4.6

VO_2MAX _(ml O_2_•kg^-1^•min^-1^)	61.0 ± 1.6	46.4 ± 1.2

### Preliminary testing

Each participant performed a VO_2MAX _test to determine position settings for the bicycle ergometer, collect baseline weight and calculate the relative work rate for the trials. VO_2MAX _tests were performed on a braked Lode Excalibur Sport bicycle ergometer (Model 911900, Lode BV, Groningen, The Netherlands) equipped with adjustable seat and handlebars, and pedals with toe clips and straps or clipless pedals. Subjects wore a heart rate monitor transmitter attached to an elastic strap (Polar Xtrainer Plus, Polar Electro Oy, Kempele, Finland) around their chest. The heart rate transmitter communicated to a wrist receiver mounted on the ergometer handlebars. Participants breathed through a Daniel's valve, and respiratory gas analysis was measured using a computer-based open-circuit system (Max-I, Physio-Dyne Instrument Corporation, Quogue, NY).

After warming up for 5 minutes at 75–100 watts, participants cycled at 150 watts for 4 minutes. Wattage increased by 50 watts every 2 minutes until 350 watts were reached, then increased 25 watts every 2 minutes until the Respiratory Exchange Ratio (RER) was greater than 1.1 and the increase in VO_2 _was less than 0.2 L•min^-1 ^or the participant could no longer continue. VO_2MAX _(ml O_2_•kg^-1^•min^-1^) was calculated by averaging the two highest 30-second interval VO_2 _values. VO_2MAX _was then used to calculate the work rate in watts at 60% VO_2MAX _for the trials using the following regression equation derived from Åstrand and Rodahl [20]:



At the completion of the VO_2MAX _test, participants were given instructions for test preparation including fasting, avoiding caffeine during the fast, and diet and exercise restrictions.

### Experimental protocol

Participants prepared for the trials by recording all food intake for two days prior and exercise three days prior to the test. They were instructed to perform only light exercise the day immediately prior to the trial and to avoid glycogen-depleting exercise within three days prior to the trial. Exercise intensity was described on a scale of 1–10 where 10 is the highest intensity and light intensity is 4 or lower. Glycogen-depleting exercise was described as exercise bouts lasting 2 hours or longer at moderate intensity of 5 or higher or 1 hour at 8 or higher. Subjects were also instructed to consume the same diet and perform consistent exercise prior to each trial. Forms were provided to record exercise during the 3 days prior and food during the 2 days prior to the trial. There were at least 4 full days but no more than 12 days between the two trials.

Treatment order was randomized so that 6 subjects consumed 2, 20-ounce bottles of a 6% carbohydrate sports drink (Drink) and 6 subjects consumed 73 g of a 100% whole grain cereal (Wheaties, General Mills, Inc., Minneapolis, MN) with 350 ml nonfat milk (Cereal) during the first trial. The amount of cereal and milk chosen were based on a typical bowl size, equal to approximately 2 servings as per the cereal box *Nutrition Facts*. The volume of drink was chosen to match the amount of carbohydrate in the cereal and milk combination. Due to the difference in the food forms, the trials could not be blinded. Instead, subjects were not informed which food they would receive during the first trial until the day of the trial.

**Figure 1:**
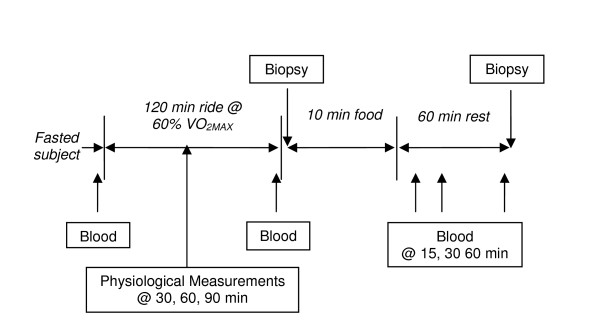
**Study protocol**.

Subjects reported to the lab in the morning at 7 am after a 12-hour fast. Food and exercise logs, and pre-exercise weight were collected. The heart rate monitor was secured against the participant's chest and the watch receiver mounted on the handlebars. Next, a 20-gauge Teflon catheter was inserted into a large forearm vein. The participant sat quietly on the ergometer for approximately 2 minutes and a resting 5 ml blood sample (Pre) and heart rate were collected (Figure 1).

Subjects warmed up for 5 minutes at 75–100 watts on the same bicycle ergometer used during the VO_2MAX _test, then cycled at a work rate equivalent to 60% VO_2MAX _for 120 minutes. During the ride, physiological measurements were collected and 250 ml of water was provided at 30, 60 and 90 minutes. These measurements included the Borg Rating of Perceived Exertion (RPE), VO_2 _and heart rate to measure exercise intensity. VO_2 _and VCO_2 _measurements (l/min) were used to calculate substrate non-protein oxidation rates (g/min) during exercise using the equations of Frayn [21] and Kaastra [22], et al.. Additionally, 5 ml blood samples were drawn immediately prior to exercise cessation (End) and 15 (Post15), 30 (Post30) and 60 (Post60) minutes after consuming the food.

After completing the 120-minute ride, the subject immediately stopped cycling, then lay supine in preparation for the muscle biopsy taken from the lateral side of the vastus lateralis. The skin was first cleaned with 10% povidone-iodine (Betadine Solution, Purdue Pharma L.P., Stamford, CT) and then anesthetized by injecting 1.5 cc of 1% Lidocaine-HCL into the skin. A 5–8 mm incision was made in the skin and subcutaneous fat, then approximately 50 mg of muscle tissue was removed using a Bergström biopsy needle (Dyna Medical, London, Ont. Canada). The first biopsy was taken within 10 minutes of exercise cessation (Post0). Subjects were then given 10 minutes to consume either Drink or Cereal. Treatments were isocarbohydrate, and Cereal provided additional energy from protein and fat (Table 2). 750 ml of water was included with Cereal to ensure similar fluid content between the treatments. After consuming the food, subjects rested upright in a chair for 60 minutes. Approximately 80 minutes post exercise (60 minutes post food or beverage), the skin was cleaned and a second muscle biopsy taken proximal from the same incision (Post60). Both biopsies were taken from the subjects' left leg during the first trial and the right leg during the second trial. Before leaving the lab, subjects were provided instructions for self care of the biopsy site. The following morning, subjects returned to the lab for examination of the biopsy site.

**Table 2:** Treatment nutrition, M ± SEM

	**Cereal**			**Drink**
Serving Size	73 g Cereal 350 ml nonfat milk 750 ml water			40 oz (1200 ml)

	*Cereal*	*Milk*	*Total Cereal & Milk*	

kcal	268	123	391	317
Carbohydrate (g)	59.0	18.0	77.0	78.5
*Per Subject (g•kg*^-1^)			*1.1 ± 0.0*	*1.1 ± 0.0*
*Range (g•kg*^-1^)			*0.9 to 1.3*	*0.9 to 1.3*

Sugars (g)	9.7	18.5	28.2	63.9

Protein (g)	7.3	12.2	19.5	0
*Per Subject (g•kg*^-1^)			*0.3 ± 0.0*	*0*
*Range (g•kg*^-1^)			*0.2 to 0.3*	*0*

Amino Acids (g)				
*Tryptophan*	*Not*	0.145	0.145	0
*Threonine*	*Available*	0.297	0.297	0
*Isoleucine*		0.544	0.544	0
*Leucine*		1.185	1.185	0
*Lysine*		0.913	0.913	0
*Methionine*		0.225	0.225	0
*Cystine*		0.446	0.446	0
*Phenylalanine*		0.526	0.526	0
*Tyrosine*		0.536	0.536	0
*Valine*		0.652	0.652	0
*Arginine*		0.261	0.261	0
*Histidine*		0.272	0.272	0
*Alanine*		0.362	0.362	0
*Aspartic acid*		0.881	0.881	0
*Glutamic acid*		2.439	2.439	0
*Glycine*		0.181	0.181	0
*Proline*		1.243	1.243	0
*Serine*		0.609	0.609	0
*Hydroxyproline*		0.000	0.000	0

Sodium (mg)	511	152	663	476

Potassium (mg)	256	565	821	183

Fiber (g)	7.3	0	7.3	0

Fat (g)	2.4	0.3	2.7	0

### Plasma analyses

At each blood collection, two glucose measurements were taken with a OneTouch Basic Glucose Meter and OneTouch Test Strips (LifeScan, Milpitas, CA) and the average recorded. The OneTouch Basic Glucose Meter was calibrated before each test session and had been previously validated with a YSI 23A Blood Glucose Analyzer (YSI Incorporated, Yellow Springs, OH). Remaining blood was split between tubes containing 10% perchloric acid (PCA) and 20 mM ethylenediamine tetraacetic acid (ETDA) and kept chilled on ice during the trial. When all samples were collected, the blood was kept chilled and centrifuged at 3000 rpm for 10 minutes, supernatant removed, then stored at -80°C until analysis. Each blood sample was analyzed for lactate (PCA) and insulin (EDTA) concentrations.

#### Lactate

Plasma lactate concentration was determined by enzymatic analysis as per Hohorst [23]. Duplicate samples were prepared by adding 1 ml glycine-hydrazine buffer (25.02 g glycine, 23.98 ml hydrazine added to dH_2_0, per liter, pH 9.2), 0.83 mg NAD, 5 μl LDH and 50 μl plasma, then incubated at 37°C for 45 min. NADH was then read with a Beckman DU640 Spectrophotometer (Coulter, Fullerton, CA) at 340 nm.

#### Insulin

Plasma insulin concentration was determined by radioimmunoassay [24]. Duplicate samples were prepared using an ImmuChem Coated Tube Insulin Kit (MP Biomedicals, LLC, Orangeburg, NY) then incubated for 18 hours at room temperature. Each tube was decanted, blotted on absorbent paper, rinsed with 4 ml de-ionized water, and decanted a second time. The remaining ^125^I was counted using a Wallac 1470 Wizard Gamma Counter (PerkinElmer Life and Analytical Sciences, Boston, MA). The curve fit algorithm was linear interpolation, point-to-point with the x-axis set to linear/log and the y-axis set to B/B_0_.

### Muscle tissue analyses

Muscle biopsy samples were trimmed of adipose and connective tissue, immediately frozen in liquid nitrogen, then stored at -80°C until analysis. The muscle tissue was analyzed for glycogen, phosphorylation (deactivation) of glycogen synthase, Akt, mTOR, rpS6 and eIF4E. These proteins are regulated by insulin and intimately involved in glycogen and protein synthesis.

#### Glycogen

Glycogen content was determined by enzymatic degradation with amyloglucosidase in a modified method of Passonneau and Lauderdale [25]. The muscle sample was weighed, digested in 1N KOH while incubated at 65–70°C for 20 minutes, mixed, then incubated for an additional 10 minutes. One hundred microliters of homogenate was added to 250 μl of 0.3 M sodium acetate (pH 4.8) then mixed. Ten microliters of 50% glacial acetic acid and 250 μl sodium acetate (containing 10 mg/ml amyloglucosidase, pH 4.8) were then added to the tubes. Tubes were sealed and incubated overnight at room temperature. The glucose reagent was prepared using a Raichem Glucose Color Reagent Kit (Hemagen Diagnostics, San Diego, CA). One hundred microliters of muscle homogenate solution and 1.5 ml of reagent were added to clean tubes then incubated for 10 minutes at 37°C. Samples were read with a Beckman DU640 Spectrophotometer (Coulter, Fullerton, CA) at 500 nm.

#### Glycogen synthase, Akt, mTOR, eIF4E, rpS6

Parameters of proteins measured by western blotting are defined as [phosphorylation site(s), antibody# (Cell Signaling Technology, Inc., Danvers, MA), sample protein weight, dilution, separation time, sodium dodecyl sulphate polyacrylamide gel electrophoresis (SDS-PAGE) matrix (Bio-Rad Laboratories, Inc., Hercules, CA)]. Exceptions are noted. Western blots were used to measure phosphorylation of glycogen synthase (Ser^641^, #9741, 50 μg, 1:2000, 75 min, 12% gel), Akt [Ser^473^, #05-736 (Upstate Cell Signaling Solutions, Lake Placid, NY), 50 μg, 1:3000, 90 min, 12% gel], mTOR (Ser^2448^, #2971, 60 μg, 1:1000, 120 min, 8% gel), eIF4E (Ser^209^, #9741, 90 μg, 1:500, 30–60 min, 12.5% gel) and rpS6 (Ser^235/236^, #2211, 50 μg, 1:1000, 50 min 12% gel). Muscle samples were weighed, then ground and homogenized with a glass pestle tissue grinder (Corning Life Sciences, Lowell, MA; Caframo Stirrer Type RZR1, Wiarton, Ont. Canada) then diluted 1:10 with a 7.4 pH chilled elongation initiation factor buffer (20 mM Hepes, 2 mM EGTA, 50 mM NaF, 100 mM KCl, 0.2 mM EDTA, 50 mM b-glycerophosphate, 1 mM DTT, 0.1 mM PMSF, 1 mM benzamidine hydrochloride hydrate and 0.5 mM sodium orthovanadate). Homogenate was centrifuged at 14,000 g for 10 minutes at 4°C, supernatant removed and stored at -80°C. Protein concentration was determined using a modification of the Lowry method [26]. Thawed aliquots of homogenized muscle were diluted 1:1 with a 6.8 pH Laemmli sample buffer (125 mM tris, 20% glycerol, 2% SDS and 0.008% bromophenol blue) [27].

Muscle proteins were separated using a SDS-Page gel, electrophoretically transferred for 15 minutes to polyvinylidene diflouride membranes (Sigma chemical Co., St. Louis, MO), and then washed in Tris-Buffered Saline (TBS) (50 mM tris, 150 mM NaCl) containing 0.06% Tween-20 (TTBS) and 5% nonfat dry milk. The membranes were incubated overnight at 4°C with the respective antibodies diluted in TTBS containing 1% nonfat dry milk. The membranes were then washed twice with TTBS and incubated for 2 hours with a secondary antibody diluted 1:2000 in TTBS containing 1% nonfat dry milk [#7074, Anti-rabbit IgG, HRP Linked Antibody (Cell Signaling Technology, Inc., Danvers, MA)]. Proteins bound to antibodies were visualized by enhanced chemiluminescence (#NEL104, Western Lightning Chemiluminescence Reagent Plus, PerkinElmer Life Sciences, Boston, MA).

Blot films were scanned and saved in TIFF on a Windows computer. ImageJ version 1.37 v software developed by the NIH was used to remove the film background and acquire two density measurements. Means of blot measurements were calculated and compared to a standard comprised of insulin-stimulated rat skeletal muscle as a percent of standard.

### Statistics

Statistical analysis was performed using SPSS 14.0 for Windows (SPSS Inc., Chicago, IL). All data are displayed as mean ± SEM. Within and between treatment analyses were performed using repeated measures ANOVA. When significance was found in plasma measurements, post hoc comparisons used a Bonferroni adjustment to reduce family-wise error. A correction factor of 2 (number of treatments) was applied to significance found in combined physiological data. Bivariate correlations were calculated using Pearson correlation coefficients. Significance was determined at p < .05.

## Results

### Physiological measurements

There were no differences between trials for all physiological measurements, so the measurements were combined for analysis (data not shown). Heart rate increased from rest and peaked 90 minutes into exercise (Rest 61.9 ± 2.9, 30 min 137.4 ± 3.3, 60 min 140.4 ± 3.3, 90 min 142.5 ± 3.5 bpm). Perceived exertion was significantly different between all three collections (30 min 11.2 ± 0.3, 60 min 12.0 ± 0.3, 90 min 12.6 ± 0.4, p < .05). Carbohydrate oxidation significantly decreased from 30 to 90 minutes (30 min 1.9 ± 0.1, 60 min 1.9 ± 0.2, 90 min 1.7 ± 0.1 g/min, p < .001) while fat oxidation significantly increased from 30 to 90 minutes (30 min 0.5 ± 0.05, 60 min 0.48 ± 0.05, 90 min 0.59 ± 0.04 g/min, p < .001).

### Plasma measurements

#### Insulin

**Figure 2:**
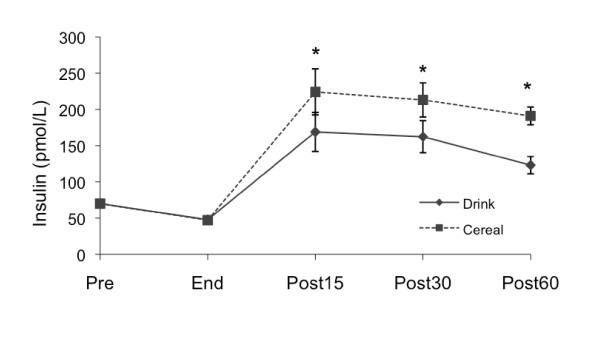
**Insulin changes by treatment**. Measured pre-exercise (Pre), at end of exercise (End), and 15, 30 and 60 minutes after supplementation (Post15, Post30 and Post60). Values are *M ± SEM*. * Significant difference between Drink and Cereal (p < .001).

Pre-exercise plasma insulin values were not significantly different between treatments (Figure 2). Plasma insulin dropped during exercise and was lowest immediately post exercise (Drink 47.8 ± 3.0, Cereal 47.2 ± 2.4 pmol/L). Insulin increased and remained higher than pre-exercise levels 60 minutes after both treatments (Drink 123.1 ± 11.8, p < .01; Cereal 191.0 ± 12.3 pmol/L, p < .001). There was a significant difference between Drink and Cereal treatment effects (p < .05); however, the post-exercise AUC was smaller for Drink as compared to Cereal (Drink 11,898.99 ± 1208.57, Cereal 15,464.79 ± 1247.92 pmol/L•60 min, p < .05). Sixty minutes after the treatment, insulin was higher for Drink compared to Cereal (p < .001).

#### Glucose

**Figure 3:**
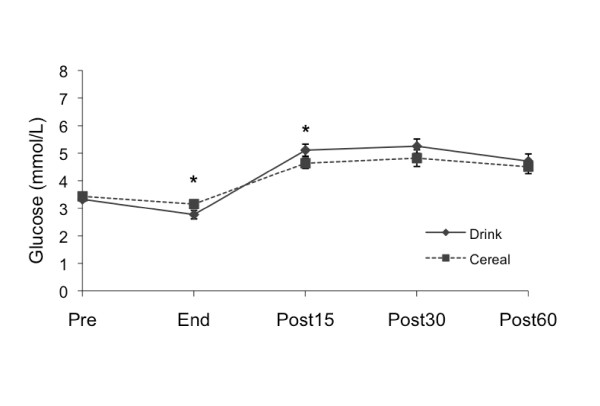
**Glucose changes by treatment**. Measured pre-exercise (Pre), at end of exercise (End), and 15, 30 and 60 minutes after supplementation (Post15, Post30 and Post60). Values are *M ± SEM*. * Significant difference between Drink and Cereal (p < .05).

Pre-exercise plasma glucose values were not significantly different between treatments (Figure 3) (Drink 4.0 ± 0.1, Cereal 4.1 ± 0.1 mmol/L). Plasma glucose dropped during exercise and was lowest immediately at the end of exercise (Drink 3.3 ± 0.2, Cereal 3.8 ± 0.1 mmol/L). Glucose increased and remained higher than pre-exercise levels 60 minutes after both treatments (Drink, 5.7 ± 0.3 mmol/L, p < .01; Cereal 5.4 ± 0.3 mmol/L, p < .05). The post-exercise AUC was higher for Drink as compared to Cereal (Drink 484.67 ± 15.57, Cereal 438.54 ± 18.31 mmol/L•60 min, p < .05). There was no significant difference between the Drink and Cereal treatment effects (p = .395).

#### Lactate

**Figure 4:**
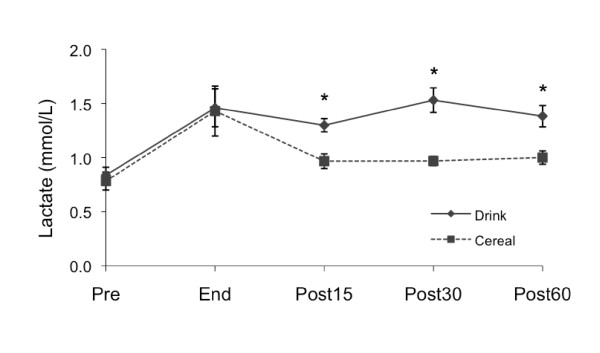
**Lactate changes by treatment**. Measured pre-exercise (Pre), at end of exercise (End), and 15, 30 and 60 minutes after supplementation (Post15, Post30 and Post60). Values are *M ± SEM*. * Significant difference between Drink and Cereal (p < .05).

Pre-exercise plasma lactate values were not significantly different between treatments (Figure 4). Plasma lactate increased during exercise (Drink 1.5 ± 0.2, Cereal 1.4 ± 0.2 mmol/L). There was a significant difference between the Drink and Cereal treatment effects (p < .05). After Drink, lactate continued to rise at 15 minutes, peaked at 30 minutes and remained significantly higher than pre-exercise levels at 60 minutes (1.3 ± 0.1, 1.5 ± 0.1, 1.4 ± 0.1 mmol/L, p < .01). After Cereal, plasma lactate dropped to pre-exercise levels at 15 minutes and remained low at 30 and 60 minutes (1.0 ± 0.1, 1.0 ± 0.0, 1.0 ± 0.1 mmol/L).

### Muscle glycogen and proteins

#### Glycogen

**Figure 5:**
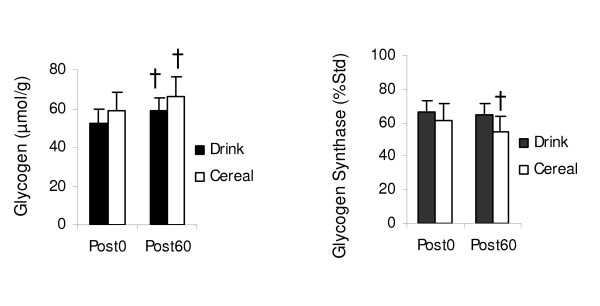
**Glycogen and glycogen synthase (Ser641) changes by treatment**. Measured immediately before supplementation (Post0) and 60 minutes after supplementation (Post60). Values are *M ± SEM*. No significant difference between treatments (glycogen, p = .682; glycogen synthase, p = 0.362). † Significant Post0 to Post60 changes glycogen (Drink, p < .05; Cereal, p < .01), glycogen synthase (Cereal, p < .05).

Muscle glycogen values did not differ between treatments immediately post exercise (Figure 5). After 60 minutes, glycogen increased significantly for both Drink (52.4 ± 7.0 to 58.6 ± 6.9 μmol/g, p < .05) and Cereal (58.7 ± 9.6 to 66.0 ± 10.0 μmol/g, p < .01); however, there was no significant difference in the rate of glycogen synthesis between treatments (p = .682).

#### Glycogen Synthase

Phosphorylation of glycogen synthase did not differ between treatments immediately post exercise (Figure 5). After 60 minutes, glycogen synthase phosphorylation decreased significantly for Cereal (61.1 ± 8.0 to 54.2 ± 7.2 %Std, p < .05) but not for Drink (66.6 ± 6.9 to 64.9 ± 6.9 %Std, p = .638); however, there was no significant difference in the mean change in phosphorylation between treatments (p = .362).

#### Akt

**Figure 6:**
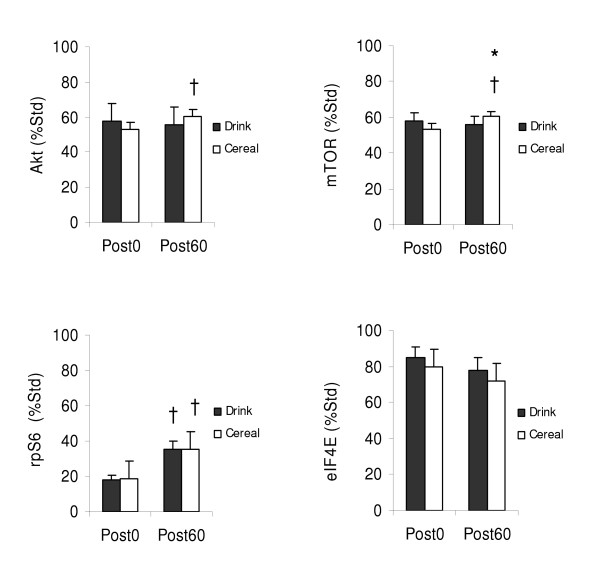
**Akt (Ser^473^), mTOR (Ser^2448^), rpS6 (Ser^235/236^), eIF4E (Ser^209^) changes by treatment**. Measured immediately before supplementation (Post0) and 60 minutes after supplementation (Post60). Values are *M *± *SEM*. No significant difference between treatments (Akt, p = .091; rpS6, p = .911; eIF4E, p = .856) except mTOR (p < .05). † Significant Post0 to Post60 changes Akt (Cereal, p < .05), mTOR (Cereal, p < .001), rpS6 (Drink, p < .001; Cereal, p < .01).

Phosphorylation of Akt did not differ between treatments immediately post exercise (Figure 6). After 60 minutes, Akt phosphorylation significantly increased for Cereal (53.2 ± 4.1 to 60.5 ± 3.7 %Std, p < .05) but was unchanged for Drink (57.9 ± 3.2 to 55.7 ± 3.1 %Std, p = .491); however, there was no significant difference in the mean change in phosphorylation between treatments (p = .091).

#### mTOR

Phosphorylation of mTOR did not differ between treatments immediately post exercise (Figure 6). After 60 minutes, mTOR phosphorylation increased for Cereal (23.0 ± 3.1 to 42.2 ± 2.5%, p < .001) but not for Drink (28.7 ± 4.4 to 35.4 ± 4.5 %Std, p = .258). There was a significant difference in the mean change in phosphorylation between treatments (p < .05).

#### rpS6

Phosphorylation of rpS6 did not differ between treatments immediately post exercise (Figure 6). After 60 minutes, rpS6 phosphorylation increased for both Drink (17.9 ± 2.5 to 35.2 ± 4.9 %Std, p < .001) and Cereal (18.6 ± 2.2 to 35.4 ± 4.4 %Std, p < .01); however, there was no significant difference in the mean change in phosphorylation between treatments (p = .911).

#### eIF4E

Phosphorylation of eIF4E did not differ between treatments immediately post exercise (Figure 6). After 60 minutes, eIF4E phosphorylation decreased but not significantly for either Drink (84.6 ± 6.4 to 78.1 ± 6.8 %Std, p = .284) or Cereal (79.8 ± 4.5 to 71.7 ± ,6.9 %Std p = .250). There was no significant difference in the mean change in phosphorylation between treatments (p = .856).

### Correlations

At 60 minutes after treatment (Post60), glycogen was correlated with phosphorylated glycogen synthase for Drink (r = .771, p < .01) and Cereal (r = .789, p < .01). At Post60, Akt was correlated with mTOR for Drink (r = .716, p < .01) but not Cereal (r = .052, p = .872). No other meaningful correlations were obtained.

## Discussion

While both a 100% whole grain cereal and nonfat milk (Cereal) and 6% carbohydrate-electrolyte beverage (Drink) increased glycogen following moderate exercise, significant phosphorylation of mTOR and AKT only occurred after Cereal. Prior research has focused on comparing the effects of carbohydrate and carbohydrate-protein post-exercise supplementation on either glycogen [13,28,29] or protein [7,14] synthesis after exercise. Our research examined the effects of readily available foods on glycogen synthesis and the phosphorylation state of proteins controlling protein synthesis after a typical cycling endurance workout.

After endurance exercise, glycogen is reduced and protein synthesis increased; however, the rate of protein degradation exceeds protein synthesis [1,7]. Recovery foods that target either glycogen storage or protein synthesis can potentially affect future exercise performance by compromising muscle protein or energy stores, respectively. Reduction in glycogen, increased glycogen synthase activity, and increased insulin sensitivity prime the muscle for glycogen synthesis post exercise; however, glucose substrate must be available to support glycogen accretion [9,19,30]. Although protein synthesis also increases after resistance and endurance exercise, without substrate, net protein balance is not positive, only less negative [6,7]. Food containing essential amino acids (EAAs) must be consumed to achieve a positive net protein balance [4] and insulin must also be present [31-33]. In our research, the carbohydrate in Drink supplied substrate for glycogen storage, but Cereal provided carbohydrate and EAAs necessary to support both glycogen and protein synthesis (Table 2).

As expected, insulin secretion during recovery was higher for Cereal compared to Drink, possibly due to the amino acids in the nonfat milk [13,34]. The plasma glucose AUC was lower after Cereal due to higher insulin and resultant increased glucose uptake by the exercised muscle, similar to other studies comparing carbohydrate-protein and carbohydrate recovery foods [13,22,35]. However, plasma lactate levels were significantly lower after Cereal compared to Drink. This drop in lactate is similar to that observed by Ivy et al. [29] after a carbohydrate-protein (80 g CHO, 28 g PRO, 6 g FAT) beverage, but not after isocarbohydrate (80 g CHO, 6 g FAT) or isocaloric (108 g CHO, 6 g FAT) carbohydrate beverages. Since plasma lactate is not a primary substrate for glycogen synthesis in the fed state [36], it is possible that a higher percentage of glucose was taken up by the muscle and stored as glycogen after Cereal rather than converted to lactate. While both treatments increased glycogen, we did not observe a difference between treatments, possibly due to the low sensitivity of the biopsy procedure or insufficient time to detect a difference.

Phosphorylation of Akt increased for Cereal but not for Drink, possibly coupled to the higher insulin levels after Cereal (Figure 6). In addition to increasing GLUT4 concentration at the cell membrane, Akt deactivates glycogen synthase kinase 3 (GSK-3), which allows activation, or dephosphorylation, of glycogen synthase [37-39]. Normally after exercise, glycogen synthase is activated to stimulate glycogen storage. As glycogen accretion occurs, glycogen synthase becomes phosphorylated, reducing glycogen synthase activity. Both Cereal and Drink increased glycogen, but compared to Drink, Cereal had lower glycogen synthase phosphorylation, suggesting that the greater Akt phosphorylation continued to stimulate glycogen synthase activity 60 minutes after Cereal despite elevated glycogen (Figure 5).

Akt also phosphorylates the mammalian target of rapamycin (mTOR), stimulating downstream phosphorylation of proteins controlling translation [40-43]. In addition to Akt, mTOR is stimulated by amino acids, particularly leucine, either directly or indirectly [33,44,45] but not aerobic exercise [15,46,47]. Unlike Drink, Cereal had a significant effect on mTOR and Akt phosphorylation (Figure 6), implying that mTOR was activated by Akt and also by the amino acids in the nonfat milk. The high correlation of Akt and mTOR for Drink but not for Cereal suggests that mTOR was directly stimulated by Akt for Drink and primarily through the alternate amino acid pathway for Cereal.

Activation of mTOR increases phosphorylation of p70^S6K^, which activates ribosomal protein S6 (rpS6), a substrate of p70^S6K^. rpS6 can also be activated by exercise through the extracellular signal-regulated kinase 1/2 (ERK1/2) through phosphorylation of p90RSK and p38 mitogen-activated protein kinase (MAPK) pathways [48-51]. The significant increases in phosphorylation of rpS6 were almost identical between Cereal and Drink (Figure 6), unlike recent human and animal studies, suggesting an exercise effect. Karlsson et al. [43] observed a slight elevation in p70^S6K ^phosphorylation and corresponding rise in rpS6 phosphorylation in men 1 hour after resistance exercise followed immediately by a placebo beverage; however, the phosphorylation of both p70^S6K ^and rpS6 were significantly higher when a branched-chain amino acid (BCAA) drink was consumed after exercise. Similar to Karlsson, our lab has observed increased rpS6 phosphorylation 45 minutes after cycling exercise after both placebo and carbohydrate-protein beverages, although rpS6 phosphorylation was significantly higher after carbohydrate-protein compared to the placebo beverage [47].

Our lab has also observed timing of rpS6 phosphorylation in rats that was highly correlated to insulin [15]. rpS6 phosphorylation was higher 30 minutes post exercise in animals given carbohydrate-protein post exercise compared to fasted, exercised controls. Interestingly, rpS6 phosphorylation was significantly increased at 90 minutes in animals that did not receive supplementation. At both time points, insulin was elevated in the respective animal groups compared to exercised controls. In the current study, we would expect the higher insulin and mTOR phosphorylation at 60 minutes after Cereal to result in higher rpS6 phosphorylation compared to Drink, but that did not occur, possibly due to the amount of supplementation provided or biopsy timing. The nearly identical increase in rpS6 phosphorylation for both Cereal and Drink suggest that these changes were due to exercise and independent of supplementation.

For translation initiation to occur, mTOR must increase phosphorylation of eukaryotic translation initiation factor 4E (eIF4E) binding protein 1 (4E-BP1), releasing eIF4E to bind to eIF4G, forming the eIF4F complex. Phosphorylation of eIF4E may be affected by phosphorylation of MAP kinase interacting serine/threonine kinase 1 and 2 (MNK1/MNK2) [52]. Ueda et al. [52] established that changes in p38 MAPK phosphorylation of MNK1 directly influenced the levels of eIF4E phosphorylation while ERK1/2 activates both MNK1 and MNK2, but primarily affects the basal level of eIF4E phosphorylation. The role of phosphorylated eIF4E in protein synthesis is unclear; while some studies have concluded that phosphorylation of eIF4E is necessary for translation [53] others have not [52,54,55]. We observed a slight, insignificant decrease in phosphorylation of eIF4E after both Drink and Cereal, with no difference between treatments (Figure 6). This lack of change in phosphorylation of eIF4E between treatments agrees with the findings of Gautsch et al. [31], who observed no change in post-exercised rats that consumed saline, carbohydrate or a mixed meal. In addition, there was no difference in phosphorylation of eIF4E between fasted-rested rats and all exercise groups, suggesting that exercise did not affect eIF4E phosphorylation.

The form of our recovery foods did not seem to affect our results, although the rate of gastric emptying would be expected to be lower for solid food versus liquid food. Reed et al. [56] did not find a difference in liquid versus solid food for glycogen synthesis but provided a larger amount of carbohydrate (1.5 g•kg^-1 ^BW) in two feedings and studied the effects after 4 hours. Differences in gastric emptying rates between solid and liquid food may further change the respective appearance rates. Also, independent of the form, the splanchnic clearance rates of EAAs are not the same, so entry of amino acids into plasma will not match the ratio contained in the food [4]. Liquid carbohydrate-protein and carbohydrate-free AA supplementation has been studied with respect to effects on protein synthesis, but direct comparisons between solid and liquid food are not as available [14,46,57]. The increase in Akt and mTOR phosphorylation, and increased glycogen in the current research, suggests that the solid whole grain cereal cleared the GI tract and was sufficiently available to the exercised muscle within 60 minutes after Cereal.

A possible limitation in our study design was the timing of the second muscle biopsy. Glycogen and protein synthesis occur at different rates, but prior research has not identified an optimal measurement strategy to detect concurrent changes. We considered 60 minutes post treatment to be sufficient to observe changes in both glycogen levels and proteins involved in translation initiation, the rate-limiting step in protein synthesis. Ivy, et al. [29] compared carbohydrate and carbohydrate-protein supplementation effects on glycogen levels after endurance exercise, testing glycogen at multiple time points using ^13^C-NMR. The glycogen accretion after a carbohydrate-protein and isocarbohydrate beverage differed between 20 and 60 minutes then converged at 2 hours. Their post exercise glycogen levels were lower and caloric content of the food higher compared to the current study, which can increase the synthesis rate during the first hour of recovery [35,58,59]. The rate of glycogen storage in the current study was suboptimal, even with supplementation, because the moderate cycling exercise did not deplete the glycogen level to support the maximal replenishment rate [58]. However, with the higher amount of active glycogen synthase and phosphorylated Akt in Cereal, we may have seen a greater amount of glycogen storage with additional supplementation and subsequent muscle biopsies.

Increased phosphorylation of proteins involved in protein synthesis has been observed within 30 minutes of both solid and liquid supplementation. Vary and Lynch [60] biopsied rested rats at 30 and 60 minutes after feeding a mixed meal. Although phosphorylation of mTOR, Akt and p70^S6K ^remained elevated at 60 minutes compared to pre-feeding levels, phosphorylation was highest at 30 minutes. Research in our lab has shown significant increase in phosphorylation of mTOR and rpS6 in humans 45 minutes after post-exercise supplementation [47]. Our results suggest that 60 minutes was sufficient to show a change in these proteins, but we may have not observed peak phosphorylation after supplementation. Further research is necessary to better understand relative timing of the phosphorylation of proteins controlling protein synthesis in humans with respect to exercise and supplementation.

## Conclusion

Although the combination of protein and carbohydrate in Cereal affected the muscle differently than the carbohydrate in Drink, glycogen accretion and phosphorylation of proteins controlling the initiation of protein synthesis, except mTOR, were similar. This suggests that readily available foods such as cereal and nonfat milk can provide post-exercise supplementation and be used in lieu of a commercially-available sports drink after moderate exercise. Cereal and nonfat milk provide a less expensive whole food option as compared to sports drinks. It also provides easily digestible and quality protein in the milk, which could promote protein synthesis and training adaptations, unlike a carbohydrate sports drink. This is a potential option for individuals who refuel at home.

## Abbreviations

4E-BP1: eukaryotic translation initiation factor 4E binding protein 1; AA: amino acid; Akt: phosphatidylinositol 3-kinase, protein kinase B, aka PKB; ANOVA: analysis of variance; AUC: area under the curve; BCAA: branched-chain amino acids; bpm: beats per minute; ^13^C-NMR: Carbon Nuclear Magnetic Resonance Spectroscopy; Cereal: whole grain cereal with nonfat milk; CHO: carbohydrate; Drink: 6% carbohydrate-electrolyte drink; EAA: essential amino acid; EDTA: ethylenediamine tetraacetic acid; eIF4E: eukaryotic translation initiation factor 4E; End: at end of exercise; ERK: extracellular signal-regulated kinase; GSK: glycogen synthase kinase; MAP: mitogen-activated protein; MAPK: mitogen-activated protein kinase; MAPK: mitogen-activated protein kinase; MNK: mitogen-activated protein kinase interacting serine/threonine kinase; mTOR: mammalian target of rapamycin; PCA: perchloric acid; Post0: immediately prior to supplement; Post15: 15 minutes after supplement; Post30: 30 minutes after supplement; Post60: 60 minutes after supplement; Pre: before exercise; PRO: protein; RER: respiratory exchange ratio; RPE: rate of perceived exertion; rpS6: ribosomal protein S6; SDS-PAGE: sodium dodecyl sulphate polyacrylamide gel electrophoresis; Ser: serine; TBS: tris-buffered saline; TIFF: tagged image file format; TTBS: tween-20 tris-buffered saline

## Competing interests

The authors declare that they have no competing interests.

## Authors' contributions

LK recruited subjects, performed VO_2MAX _tests, coordinated trial personnel, performed lactate assay, performed all statistical analysis and wrote document. ZD handled blood, assisted during VO_2MAX _tests and trials, supervised assays, ran insulin assay, made reagents used in assays. BW handled blood, assisted during trials, performed glycogen assay. DH performed Western blots. YHL performed Western blots. JI defined the protocol, wrote and acquired grant, performed muscle biopsies, directed muscle tissue assays, reviewed and wrote portions of document. All authors read and approved the final manuscript.
